# Minimally Adequate Mental Health Treatment and Mortality in Primary Care Older Adults With Depression and Anxiety

**DOI:** 10.1093/geroni/igab046.3133

**Published:** 2021-12-17

**Authors:** Helen-Maria Vasiliadis, Catherine Lamoureux-Lamarche, Sébastien Grenier, Pasquale Roberge

**Affiliations:** 1 Université de Sherbrooke, Université de Sherbrooke, Quebec, Canada; 2 Université de Montréal, Institut universitaire de gériatrie de Montréal, Quebec, Canada

## Abstract

Receipt of quality mental health (MH) care can influence mortality. Given the scarce literature on the topic, the aim was to assess the 3-year risk of mortality in older adults (OA) associated with receiving adequate MH treatment for depression/anxiety in an epidemiologic context. The study sample included 358 OA with depression/anxiety recruited in primary care practices and followed prospectively for 3 years. Mortality was assessed from vital statistics data. Adequate care was based on receipt of pharmacotherapy, follow-up care and psychotherapy. Propensity score analysis was carried out where the inverse probability (IPW) of receiving adequate treatment was calculated. Time to event analyses with IPW was used to assess the effect of receipt of adequate MH treatment on the risk of mortality controlling for individual and health system factors. The results showed that receipt of adequate MH treatment reduced the risk of mortality (HR0.44; 95% CI: 0.22 – 0.99). Individual factors that increased mortality were male sex, being single, reduced functional status and cognitive functioning, # physical disorders, current smoking; while exercise reduced risk. Health system factors such as past # of hospitalizations increased the risk; while # of emergency department visits and continuity of care reduced mortality. Finally, treating depression/anxiety with minimal follow-up care and pharmacotherapy or psychotherapy has a significant impact on reducing mortality in OA. Primary care physicians should recognize the important potential impact of years of lives saved when providing quality MH care to OA.

